# Ceo_2_ Based Catalysts for the Treatment of Propylene in Motorcycle’s Exhaust Gases

**DOI:** 10.3390/ma7117379

**Published:** 2014-11-17

**Authors:** Phuong Thi Mai Pham, Thang Le Minh, Tien The Nguyen, Isabel Van Driessche

**Affiliations:** 1 Department of Organicsynthesis and Petrochemistry, Faculty of Chemical Technology, Hanoi University of Technology, 1 Dai Co Viet, Hanoi 10000, Vietnam; 2 Sol-gel Centre for Research on Inorganic Powders and Thin films Synthesis (SCRiPTS), Department of Inorganic and Physical Chemistry, Ghent University, Krijgslaan 291-S3, 9000 Gent, Belgium

**Keywords:** ceria, zirconia, tin oxide, cobalt oxide, oxidation, propylene, oxides, exhaust gases

## Abstract

In this work, the catalytic activities of several single metallic oxides were studied for the treatment of propylene, a component in motorcycles’ exhaust gases, under oxygen deficient conditions. Amongst them, CeO_2_ is one of the materials that exhibit the highest activity for the oxidation of C_3_H_6_. Therefore, several mixtures of CeO_2_ with other oxides (SnO_2_, ZrO_2_, Co_3_O_4_) were tested to investigate the changes in catalytic activity (both propylene conversion and CO_2_ selectivity). Ce_0.9_Zr_0.1_O_2_, Ce_0.8_Zr_0.2_O_2_ solid solutions and the mixtures of CeO_2_ and Co_3_O_4_ was shown to exhibit the highest propylene conversion and CO_2_ selectivity. They also exhibited good activities when tested under oxygen sufficient and excess conditions and with the presence of co-existing gases (CO, H_2_O).

## 1. Introduction

The complete oxidation of propylene, one component in automobile exhaust gases, has been studied by many researchers. The most popular used catalysts are noble metals such as Pt, Pd... The catalysts based on noble metals possess pollutant conversions higher than 90% [1,2,3,4]. Other catalysts such as perovskites, metallic oxides have also been investigated [5,6,7,8,9,10]. Amongst them, CeO_2_ has been widely used because it has a high oxygen storage/release capacity. However, with increasing temperature (>850 °C), CeO_2_ readily sinters, resulting in a deactivation of the catalyst [11]. Recently, some authors paid attention on the use of ceria–zirconia mixed oxides due to their good oxygen storage capacity (OSC) and their enhanced stability against thermal sintering [12,13,14]. A large number of investigations have shown that the addition of ZrO_2_ to CeO_2_ forms a CeO_2_-ZrO_2_ solid solution that could promote the bulk performance of CeO_2_ since it increases thermal stability, facilitates mobility and diffusion of bulk oxygen [15]. The addition of ZrO_2_ to CeO_2_, therefore, improves conversions of CO and hydrocarbon (HC) under reduction conditions or improves conversion of NO under oxidation conditions [16,17]. However, no agreement has been obtained about which Ce/Zr ratio could result in the highest activity of ceria–zirconia catalysts in the treatment of propylene. 

Besides CeO_2_, some other metallic oxides (cobalt oxide, manganese oxide, nickel oxide, tungsten oxide and ferrite oxide) were also investigated in detail [18,19,20,21,22,23,24,25,26]. The complete oxidation of hydrocarbons may reach approximately 100% at low temperatures by using combinations of oxide catalysts.

Since metallic oxides are normally the cheapest and most convenient catalysts for the treatment of automobile exhaust gases, our research aims to find out some candidates with high ability to treat propylene, a main pollutant component in the motorcycle’s exhaust gases. Therefore, several known metallic oxides (SnO_2_, TiO_2_, Al_2_O_3_, V_2_O_5_, Co_3_O_4_, NiO, CeO_2_, ZrO_2_, MnO_2_, ZnO, CuO) for the treatment of exhaust gases were investigated under same reaction conditions to select potential candidates. Since CeO_2_ possesses many remarkable properties as mentioned previously, the mixtures of CeO_2_ with some promising metal oxide candidates will be studied to see the influence of mixing on the catalytic activity. Different from other investigations in the literature, the screening of potential catalysts in this work was performed under oxygen deficient condition, which is close to the real high speed operating conditions of motorcycles. It was also expected that if a catalyst exhibits good activity for complete oxidation under oxygen deficient condition, it will exhibit even better activity under other oxygen sufficient conditions. Although the researches on the complete oxidation of hydrocarbons under oxygen deficient condition has not been adequately paid attention in the literature, in 1998 Lee *et al.* [27] investigated thoroughly the complete oxidation of CO and propylene under different oxygen deficient conditions. However, the authors studied Pd/Al_2_O_3_ catalysts, which is noble catalyst. Therefore, it will be interesting if one would compare activity of mixed metal oxide catalysts applied in this work with that of the noble catalysts at oxygen deficient conditions. It was also aimed that the optimal mixtures found from this research will be applied for the treatment of other components in the exhaust gases (other hydrocarbons, NO_x_, CO) in the followed papers.

## 2. Experimental

The work uses several commercial metallic oxides: SnO_2_ (99%, Merck, West Point, PA, USA), TiO_2_ (99.5%, Merck), Al_2_O_3_ (100%, Merck), V_2_O_5_ (99%, Merck). To prepare other oxides (CeO_2_, ZrO_2_, Co_3_O_4_, NiO, MnO_2_, ZnO and CuO), a sol-gel method was used. It was previously shown that this sol-gel method leads to the formation of very pure and homogeneous catalyst powders exhibiting high surface area [28]. More details on the stabilization of metal ions with coordinating species and their analytical study can be found elsewhere [29,30,31,32].

The starting solutions for sol-gel precursors with concentrations of 0.125 M were prepared from Ce(NO_3_)_3_·6H_2_O (98.5%, Merck), ZrOCl_2_·8H_2_O (99.0%, Merck), Co(NO_3_)_2_·6H_2_O (99.0%, Merck), Ni(NO_3_)_2_·6H_2_O (99.0%, Merck), Mn(NO_3_)_2_ solution (58 wt%, Merck), Zn(NO_3_)_2_·4H_2_O (98.5%, Merck), Cu(NO_3_)_2_·3H_2_O (99.5%, Merck). 10 wt% citric acid solution prepared from citric acid monohydrate—C_6_H_8_O_7_·H_2_O (99.5%, Merck) was added as a complexing agent. The molar ratio of citric acid to metal ion was 2.6. The gelification was carried out at 60 to 80 °C until transparent gels were obtained. The gels were then dried at 120 °C for 2 h. The obtained powders were calcinated at 500 °C and 600 °C in air for 3 h.

Mixtures of CeO_2_ with ZrO_2_ or Co_3_O_4_ were also prepared by the above described sol-gel synthesis. During the gelation, if precipitation occurs, an appropriate amount of concentrated HNO_3_ solution was added.

X-ray powder diffraction (XRD) patterns of the catalysts were recorded with a D8 Bruker Advanced diffractometer (Bruker, Karlsruhe, Germany). The specific surface areas of the samples were measured at 77 K by the Brunauer–Emmett–Teller (BET) method using N_2_ adsorption/desorption on an ASAP 2010 and a Gemini VII Micromeritics apparatuses (Micromeritics, Norcross, GA, USA). The morphology of the catalysts was examined on a Hitachi X4800 scanning electron microscope. Temperature Programmed Reduction Hydrogen (TPR-H_2_) profiles of the catalysts were measured with a AutoChem 2920 II—Micromeritics device (Micromeritics, Norcross, GA, USA).

Catalytic activities were measured on a micro reactor with an internal diameter of 0.4 cm. Prior to use, the catalyst were pressed, ground and sieved into 250–300 μm particles, 0.1 g of the obtained catalyst was used for each reaction. The total reactant gas flow was 80 mL/min, the gas hourly space velocities (GHSV) was 76,000 h^−1^, the volume compositions of the reactant gas flow were 2.5% C_3_H_6_, 2.5% O_2_, 95% N_2_. In order to investigate the influences of co-existing gases and oxygen concentrations on the reactions, other compositions were also adjusted: 0.9% C_3_H_6_, 4.1% O_2_, N_2_ balance; 0.9% C_3_H_6_, 5% O_2_,, N_2_ balance; 0.9% C_3_H_6_, 0.3% CO, 5% O_2_, N_2_ balance. To evaluate the influence of H_2_O, the reactant gases were flowed through a water bubbler at 25 °C. The water concentration was calculated as 2% by simulation method with a Hysys program. The reaction temperatures ranged from 200 °C to 500 °C which were measured using a thermocouple attached at the position of the catalyst bed inside an electric furnace. Analysis of propylene, oxygen, CO_2_, CO and oxygenate products was performed using an on-line Focus–Thermo Scientific gas chromatograph with a thermal conductivity detector (TCD). The data were obtained when the reaction reached to the stable state, *i.e.*, from 60 min after the starting of the reactant flows. The data were stable for at least 8 hours on stream with the continuous reactant flows and during at least three cycles of the same catalytic tests.

Pollutant concentrations were measured by a specific driving cycle ECE R40 (Economic Commission for Euro Regulation 40—Emission of gaseous pollutants of motorcycles, max speed: 50 km/h, average speed: 18.7 km/h, total time: 780 s, path length: 4.052 km). The compositions of motorcycles’ exhaust gases were analyzed by a CEBII gas analyzer (Austria AVL-Germany Pierburg). CO, CO_2_ is analyzed by a non-dispersive infrared (NDIR) analyzer. NO_x_ is analyzed by a chemiluminescence detector (CLD). The oxygen concentrations in the exhaust gases were measured under different operating conditions: idle, medium speed and high speed. Furthermore, the compositions of hydrocarbons in the exhaust gas were analyzed by a GC-MS 2010 Shimadzu (Kyoto, Japan) and a GC-FID Thermo Electron (San Jose, CA, USA).

## 3. Results and Discussion

### 3.1. Composition of Motorcycle Exhausts Gases 

To establish a suitable composition of reactants which reflect the composition of motorcycles’ exhaust gases, the compositions of the exhaust gases from different kinds of motorcycles in Hanoi, Vietnam were measured. The largest portions are CO_2_ and O_2_ which ranges from 4,000 to 120,000, 18,172 to 120,465 ppm (by volume), respectively. Due to the motorcycles in operation in Hanoi has been used for long, the compositions of HC (5000–45,000 ppm) and CO (5,000–80,000 ppm) are quite high. In the addition, the concentration of NO_x_ is the lowest as 0–4000 ppm. Since ECE R40 driving cycle lasted for 780 s, the concentrations of each component were examined for every 5 s. The data presented the range of instantaneous concentrations at every measurement. These data were obtained when tested ten motorcycles (Dream, Wave, Nouvo, Jupiter), which were new and used for about 50,000 km. Therefore, large ranges of HC, CO, CO_2_ are due to different speed applied in the ECE R40 driving cycle, different situation of the engines (old or new), which may be representative for the compositions of general working motorcycles. Compared to the data of normal engine exhaust gases obtained from literature [33], the composition of toxic gases (CO, HC) in motorcycles’ exhaust gases shown here are much higher since the motorcycles in operation in Hanoi has been used for long. Thus, the requirement for treatment is stringent. The investigation of oxygen concentration at different operating conditions (idle, medium and high speed conditions) showed that at high speed condition, the oxygen concentration is minimum (1.8 vol%). It is clear that under that operating conditions, oxygen in the exhaust gases is deficient for the complete oxidation of HC and CO. The investigation of HC in the different exhaust gases by GC-MS show that they include methyl-cyclopentane (1200–10,800 ppm), 2,3-dimethyl pentane (about 4500 ppm), toluene (500–7200 ppm), benzene (about 3000 ppm), methane (1,250–10,000 ppm), propylene (1000–8550 ppm), methanol (about 4500 ppm), ethanol (1000–17,000 ppm), acetaldehyde (2000–4500 ppm) and a few other minor gases. Amongst them, propylene was detected with high concentration. Therefore, in our work, C_3_H_6_ was chosen as the pollutant component for the treatment. Based on the real compositions of C_3_H_6_ and O_2_ in the exhaust gases, the concentration of the reactant gases was chosen as 2.5 vol% C_3_H_6_, 2.5 vol% O_2_ and 95 vol% N_2_, which is the oxygen deficient condition for the oxidation of HC. The selection of C_3_H_6_ as the object for the treatment is mainly to make a model to test the oxidation ability of the catalysts. Good catalysts found from this investigation will then be tested for the treatment of real exhaust gases, which include also other kinds of volatile organic compounds.

### 3.2. Characterization and Catalytic Activities of Several Single Metallic Oxides 

Several single metallic oxides which were found pure from XRD investigation were tested under the same reaction conditions as mentioned previously. Since the reaction condition was oxygen deficient, oxygenate products and CO were also examined during the reaction. CO_2_ selectivity was calculated based on the reaction products detected by GC, which are CO_2_, CO, formic acid, acrolein, acetaldehyde, and ethanol. In fact, more oxidation products may still exist with low concentrations and the real selectivity may be lower. However, the CO_2_ selectivity calculated here is still suitable to compare catalytic activities of the catalysts working under the same conditions and being calculated by the same way.

Propylene conversion and CO_2_ selectivity of the investigated oxides are shown in Table 1 and Table 2, respectively. Amongst these catalysts, NiO was only studied at temperatures below 350 °C due to its low thermal resistance. At higher temperatures, it was observed that NiO particles were broken-up, resulting in blocking of the reactor. The same observation was also seen with MnO_2_ at temperatures above 450 °C. However, the instability of these catalysts at high temperatures was only due to mechanical reason since TGA/DTA and XRD results indicated no change in phase compositions of the samples at the reaction temperatures. Therefore, their mechanically instability will not significantly influence their catalytic activities.

The results from Table 1 show that the propylene conversions of the catalysts almost reach to a maximum value at a certain temperature. MnO_2_ and Co_3_O_4_ exhibit high conversions from 250 °C on, whereas NiO shows a high conversion at 350 °C, V_2_O_5_ and CeO_2_ at 400 °C and SnO_2_, ZnO at 450 °C. For Al_2_O_3_, ZrO_2_ and CuO, propylene conversion still stays very low even at high temperatures up to 500 °C. Co_3_O_4_ and NiO catalysts result in the highest propylene conversion. MnO_2_ and CeO_2_ exhibited only a slightly lower conversion.

**Table 1:** Propylene conversion (%) of several oxides at different reaction temperatures.

Samples	200 °C	250 °C	300 °C	350 °C	400 °C	450 °C	500 °C
Al_2_O_3_ (118 m^2^/g)	2.67	2.34	2.25	2.42	2.77	3.78	4.69
CeO_2 _ (33 m^2^/g)	2.85	3.30	13.09	15.31	22.41	24.52	25.44
Co_3_O_4_ (11 m^2^/g)	5.69	28.78	29.42	29.74	30.00	32.97	41.67
NiO (11 m^2^/g)	5.68	4.70	6.95	29.45	-	-	-
SnO_2_ (9 m^2^/g)	2.91	2.48	3.27	4.47	8.28	16.87	17.67
TiO_2_ (54 m^2^/g)	1.95	3.05	2.70	4.35	11.68	17.06	18.18
V_2_O_5_ (4 m^2^/g)	2.93	2.57	4.21	12.25	22.69	19.58	19.73
ZrO_2_ (52 m^2^/g)	2.92	2.04	2.32	2.59	3.01	4.14	6.02
MnO_2_ (6 m^2^/g)	5.40	21.77	22.72	23.48	23.17	24.22	-
ZnO (14 m^2^/g)	3.99	3.75	3.71	4.11	8.86	23.16	33.04
CuO (2 m^2^/g)	0.29	5.17	5.82	6.73	6.55	8.08	9.36

**Table 2:** CO_2_ selectivity (%) of some metal oxides at different reaction temperatures.

Samples	250 °C	300 °C	350 °C	400 °C	450 °C	500 °C
Al_2_O_3_	-	-	-	-	24.4	46.72
CeO_2_	-	100	86.54	89.07	89.02	89.18
Co_3_O_4_	94.71	94.38	93.95	80.56	76.16	39.31
NiO	100	100	91.78	-	-	-
SnO_2_	-	-	34.68	71.39	70.47	71.64
TiO_2_	-	-	41.61	39.80	30.66	21.83
V_2_O_5_	-	29.61	20.22	29.09	31.18	34.08
ZrO_2_	-	-	-	-	24.44	36.63
MnO_2_	98.47	99.32	90.54	92.03	90.86	-
ZnO	-	-	22.51	31.11	46.52	76.07
CuO	-	-	25.88	17.00	34.05	43.41

These catalysts also possess rather high CO_2_ selectivity as seen from Table 2. However, CO_2_ selectivity of Co_3_O_4_ decreases dramatically at high temperatures due to the formation of more CO (selectivity at 500 °C is 61%). Oxygenated products were observed in the oxidation of propylene on all investigated catalysts but especially found in the reactions with V_2_O_5_, SnO_2_, TiO_2_, ZnO since these catalysts are well known catalysts for partial oxidation of hydrocarbons. Oxygenated products may also be formed when using CuO and ZrO_2_ but because CuO and ZrO_2_ exhibited low conversions, the amount of formed oxygenated products may be too low to be detected. For V_2_O_5_, SnO_2_, TiO_2_, ZnO, CO_2_ selectivity was low at 350–400 °C because these temperatures are optimal for partial oxidation to form oxygenate products (selectivity of oxygenate products may reach about 30%). When increasing temperature, CO_2_ selectivity on these catalysts increased significantly as the formed oxygenated products were also completely oxidized to CO_2_. Amongst the investigated catalysts, CeO_2_, and especially MnO_2_ exhibit quite constant and highest CO_2_ selectivity at all examined temperatures. When BET surface areas (Table 1) were taken into account, it is clear that high surface area oxides such as Al_2_O_3_, TiO_2_, ZrO_2_ exhibited low activity, they are only suitable to be supports. Oppositely, low surface area oxides such as MnO_2_, Co_3_O_4_, NiO, CeO_2_ exhibited good activity and suitability to act as active phases. Therefore, if surface areas of highly active oxides are increased, the activities of the catalysts will be improved.

In general, MnO_2_, CeO_2_ and Co_3_O_4_ are the most promising catalysts to convert propylene under oxygen deficient conditions. CeO_2_ has a high ability to convert propylene with high CO_2_ selectivity at all investigated reaction temperatures due to a high OSC as discussed in literature [16]. Co_3_O_4_ has a high propylene conversion at low temperatures but also a low CO_2_ selectivity at high temperatures. MnO_2_ shows high activity for both propylene conversion and CO_2_ selectivity but it is mechanically unstable at high temperatures. Therefore, in the following investigation, catalyst mixtures containing CeO_2_ will be focused on. 

### 3.3. Characterization and Catalytic Activities of Mixtures of CeO_2_

The catalytic activity of mixtures of CeO_2_ and SnO_2_ with different compositions was investigated since it was expected that the addition of the high conductive semiconductor SnO_2_ on the highly active catalyst CeO_2_ will improve the reaction due to the increase of the available lattice oxygen, which may act as an oxidizing agent at high temperatures. However, no development of catalytic activity has been observed with the mixtures of CeO_2_ and SnO_2_.

Although catalytic activity of ZrO_2_ was low as seen from the previous section, the catalytic activities of CeO_2_–ZrO_2_ chemical mixtures were also studied since the literature reports that ZrO_2_ is able to modify the sub-lattice oxygen in the CeO_2_–ZrO_2_ mixed oxides, generating defective structures and highly mobile oxygen atoms in the lattice which can be released even at moderate temperatures [12,13,34]. Therefore, the activities of these chemical mixtures are expected to be increased. Moreover, ZrO_2_ exhibited higher surface area than CeO_2_ as shown in Table 1, thus, the addition of ZrO_2_ may help to increase surface area of the catalysts to improve their activities.

**Figure 1:**
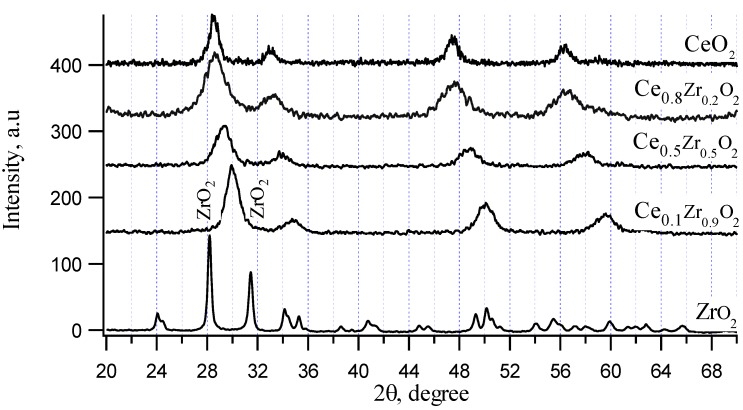
X-ray patterns of CeO_2_-ZrO_2_ chemical mixtures.

XRD patterns of CeO_2_–ZrO_2_ chemical mixtures are shown in Figure 1. CeO_2_ exhibits a cubic structure (a, b, c parameter is 5.4 nm) represented by 2θ = 28.6° and 33.1°, ZrO_2_ calcined at 550 °C (temperature lower than 1170 °C) exhibits a monoclinic structure represented by 2θ = 28.2° and 31.6°. The evidence of solid solution formation is given by the shift of ceria reflections to higher values for Ce_0.8_Zr_0.2_O_2_ and Ce_0.5_Zr_0.5_O_2_ samples, where Zr (atomic radius is 160 pm) replaced for Ce (atomic radius is 181.8 pm) in the cubic structure of CeO_2_. In the case of sample with higher Zr ratio, it has been reported in the literature [35] that a compound Ce_0.25_Zr_0.75_O_2_ shows the strongest XRD reflection at 2θ = 30°. In our work, the sample Ce_0.1_Zr_0.9_O_2_ also showed a cubic structure with the strongest XRD reflection at 2θ = 30°. Besides, single ZrO_2_ monoclinic phase still existed, which represented by small peaks at 2θ = 28.2° and 31.6° as observed in XRD pattern of this sample. Here, it should be noticed that ZrO_2_ also exist as cubic structure (a, b, c parameter is 5.1 nm) when synthesized at high temperature (more than 2370 °C). Thus, cubic structure of ZrO_2_ is very close to that of CeO_2_ with almost the same parameters. The strongest XRD reflection of this cubic ZrO_2_ is at 2θ = 30°. Therefore, in the presence of CeO_2_, ZrO_2_ cubic structure may already be formed at low temperature because a few percentage of other oxide may stabilize cubic ZrO_2_. Since the structure of cubic ZrO_2_ and cubic CeO_2_ are very similar (very close a, b, c parameters and strongest XRD reflections) and the content of CeO_2_ in Ce_0.1_Zr_0.9_O_2_ sample was small, XRD patterns of Ce_0.1_Zr_0.9_O_2_ sample looked like that of pure cubic CeO_2_ with 2θ shifted to 30° but the existed phase could be mainly assigned for cubic ZrO_2_.

All investigated CeO_2_–ZrO_2_ chemical mixtures possess surface areas around 50 m^2^/g, which are almost equal to those of pure ZrO_2_ (52 m^2^/g). Pure CeO_2_ possesses a little lower surface area (33 m^2^/g).

**Figure 2:**
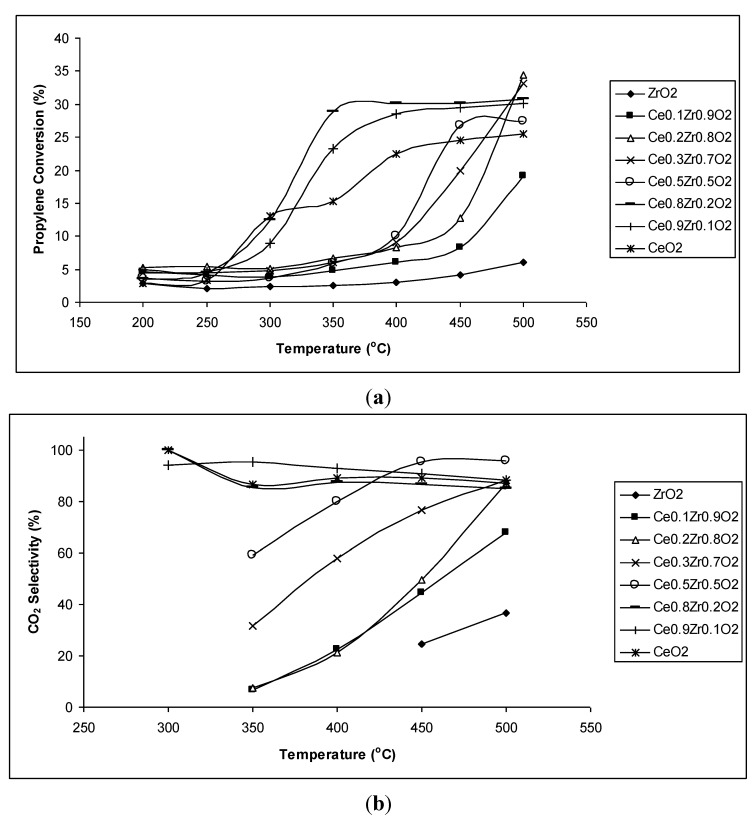
(**a**) Propylene conversion (%) and (**b**) CO_2_ selectivity (%) of CeO_2_–ZrO_2_ chemical mixtures at different reaction temperatures.

The propylene conversions of CeO_2_–ZrO_2_ chemical mixtures presented in Figure 2a indicate that the samples containing a small content of ZrO_2_ (10%–20% mol, such as Ce_0.9_Zr_0.1_O_2_ and Ce_0.8_Zr_0.2_O_2_) exhibit high propylene conversions, even at low temperature (350 °C). Their propylene conversions are even higher than that of the most active pure components CeO_2_, thus, a synergy effect has occurred. Meanwhile, the samples with higher ZrO_2_ content only reach high conversions at high temperatures (450 °C, 500 °C). Their conversions, however, are higher than that of the least active pure component (ZrO_2_). 

Figure 2b shows CO_2_ selectivity of CeO_2_–ZrO_2_ chemical mixtures at different reaction temperatures. It can be seen that CO_2_ selectivity of all CeO_2_–ZrO_2_ chemical mixtures is much higher than that of the least active pure component (ZrO_2_). However, only samples with low ZrO_2_ content (Ce_0.9_Zr_0.1_O_2_ and Ce_0.8_Zr_0.2_O_2_) exhibit comparable and quite constant high CO_2_ selectivity. The high ZrO_2_ content samples possess low CO_2_ selectivity at temperatures ranging from 350–400 °C since these temperatures were favorable for partial oxidation to form oxygenated products. Combining propylene conversion and CO_2_ selectivity, it is clear that Ce_0.9_Zr_0.1_O_2_ and Ce_0.8_Zr_0.2_O_2_ samples exhibit the highest activity for the complete oxidation of propylene into the nontoxic product CO_2_. The activity of these catalysts even increased significantly when the reaction was performed under the oxygen sufficient condition (molar ratio of C_3_H_6_/O_2_ was 1/4). The propylene conversions under the oxygen sufficient condition at 500 °C on Ce_0.9_Zr_0.1_O_2_ and Ce_0.8_Zr_0.2_O_2_ catalysts were 77.10% and 85.42%, respectively. The CO_2_ selectivity under this condition was above 97%. The catalytic activity of Ce*_x_*Zr_1−*x*_O_2_ for the oxidation of propylene under oxygen excess conditions have been reported by D. Homsi *et al.* [36], who found no enhancement of activity of the Ce_0.75_Zr_0.25_O_2_ solid solution compared to that of pure CeO_2_. That result is a bit different with the result of this work as Ce_0.8_Zr_0.2_O_2_ exhibited higher activity than that of pure CeO_2_. The reason may be the different synthesis methods, which may result in different properties of the final products. The catalyst in our work was prepared by solgel synthesis, which may allow better access of zirconium into the structure of ceria, leading to the increase of mobile oxygen as seen from TPR-H_2_ results (Table 3). Oppositely, the catalysts in D. Homsi’s work were prepared by precipitation, the method allows worse mixing of components than sol-gel method. Moreover, surface area of our Ce_0.8_Zr_0.2_O_2_ was higher than that of our CeO_2_ while it is opposite in D. Homsi’s work. Therefore, both catalytic activity and reduction ability of Ce_0.75_Zr_0.25_O_2_ was not higher than that of CeO_2_ in D. Homsi’s work. Besides, different reaction conditions (oxygen deficient in our work and oxygen excess in D. Homsi’s work) may also influenced since the advance properties of a solid solution will be more presented under oxygen deficient conditions.

**Table 3:** Quantity of hydrogen consumed (mL/g) at different reduction peaks in TPR-H_2_ profiles of pure CeO_2_, ZrO_2_, Co_3_O_4_ and some potential CeO_2_ chemical mixtures.

	Samples	CeO_2_ (33 m^2^/g)	ZrO_2_ (52 m^2^/g)	Co_3_O_4_ (11 m^2^/g)	Ce_0.9_Zr_0.1_O_2_ (42 m^2^/g)	Ce_0.8_Zr_0.2_O_2_ (46 m^2^/g)	20% CeO_2_–80% Co_3_O_4_ (45 m^2^/g)
Temp. (°C)							
279		–	–	–	–	–	28.97
316		–	–	–	9.55	–	–
364		–	–	–	–	–	12.21
375		–	–	–	2.84	–	–
430		–	–	250.54	–	–	–
474		4.62	–	–	–	–	–
503		–	–	–	–	–	101.25
531		–	–	–	18.16	–	–
536		–	–	–	–	10.29	–
580		–	–	39.25	–	–	–
623		–	0.91	–	–	–	–
625		–	–	–	–	12.04	–
642		–	4.36	–	–	–	–
688		–	–	–	2.89	–	–
694		6.23	–	–	–	–	–
**Total**		**10.85**	**5.27**	**289.79**	**33.44**	**22.33**	**142.43**

**Figure 3:**
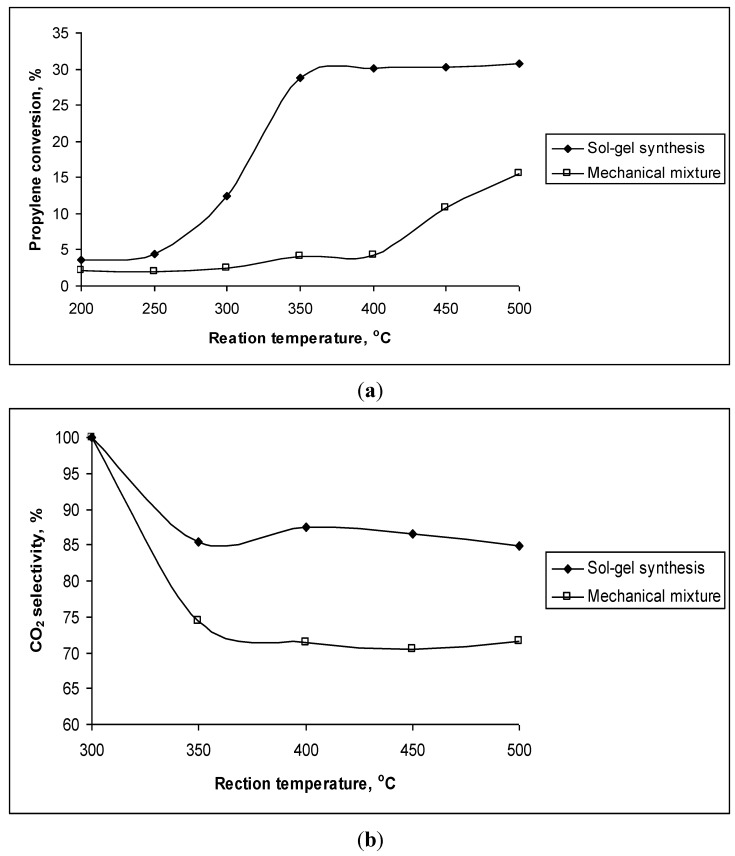
(**a**) Propylene conversion (%) and (**b**) CO_2_ selectivity (%) of the mixture containing 80% mol CeO_2_ synthesized by mechanical mixing and sol-gel method (Ce_0.8_Zr_0.2_O_2_) at different reaction temperatures.

The reason for the enhancement in catalytic activity of Ce_0.9_Zr_0.1_O_2_ and Ce_0.8_Zr_0.2_O_2_ samples, thus, may be the formation of the solid solution Ce_1−*x*_Zr*_x_*O_2_ in the chemical mixtures of CeO_2_–ZrO_2_. To prove this assumption, the CeO_2_–ZrO_2_ mechanical mixture containing 80% mol CeO_2_ was also tested for the reaction (Figure 3). This mechanical mixture showed the presence of only single CeO_2_ and ZrO_2_ phases but not solid solution. It can be observed that both propylene conversion and CO_2_ selectivity of the mechanical sample are much lower than that of the sol-gel prepared sample, where the formation of a solid solution Ce_0.8_Zr_0.2_O_2_ was detected. 

However, the sol-gel samples with high content of ZrO_2_ did not show improvement of activity. Thus, it may be assumed only a little change in the structure of Ce_1−*x*_Zr*_x_*O_2_ solid solution compared to that of CeO_2_ helps to increase catalytic activity. For other Ce_1−*x*_Zr*_x_*O_2_ solid solution (*x* > 0.5), where the structure shows a big shift of ceria reflections to higher values, the increase of catalytic activity will not happen. The formation of a solid solution with a little replacement of Zr ions to Ce ions may increase the catalytic activity since this replacement may result in appropriate vacancies inside the bulk structures of the catalysts, which increases mobility of oxygen transported inside the bulk structures, *i.e.*, also increases the OSC of the catalyst. An evidence of the increase of OSC of the Ce_1−*x*_Zr*_x_*O_2_ catalyst with low ZrO_2_ content compared to pure oxides was estimated based on TPR-H_2_ profiles of the pure oxides and chemically mixed samples (Table 3). The consumed H_2_ quantities on the solid solutions of CeO_2_ and ZrO_2_ (Ce_0.9_Zr_0.1_O_2_ and Ce_0.8_Zr_0.2_O_2_) were much higher than those on pure oxides (CeO_2_ and especially ZrO_2_). At the same time, the temperature of hydrogen reduction decreased significantly on the Ce_0.9_Zr_0.1_O_2_ catalyst (the lowest reduction temperature of Ce_0.9_Zr_0.1_O_2_ sample is only 316 °C while that of CeO_2_ is 474 °C and of ZrO_2_ is 623 °C), therefore, the catalyst reached to high activity at lower temperature. However, the fact that the catalytic activity of Ce_0.9_Zr_0.1_O_2_ was a little lower than that of Ce_0.8_Zr_0.2_O_2_ although the consumed hydrogen amount of Ce_0.9_Zr_0.1_O_2_ was a little higher than that of Ce_0.8_Zr_0.2_O_2_ is a bit non logical. Here, the influence of surface area might be a reason as surface area of Ce_0.8_Zr_0.2_O_2_ was higher than that of Ce_0.9_Zr_0.1_O_2_, which may help to expose more active sites.

**Figure 4:**
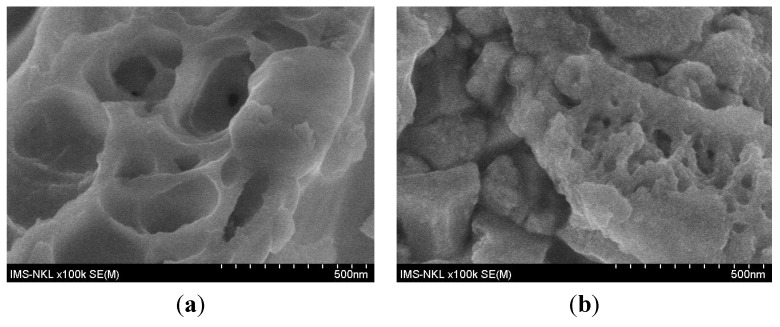
SEM images of Ce_0.8_Zr_0.2_O_2_ sol-gel sample (**a**) before and (**b**) after reaction.

Figure 4 shows the morphology change of the Ce_0.8_Zr_0.2_O_2_ sol-gel sample before and after reaction (24 h on stream). Before the reaction, the sample possesses many pores. After reaction, almost all pores are encapsulated. The reason may be the exothermicity of complete oxidation of C_3_H_6_ and the formation of coke.

**Figure 5:**
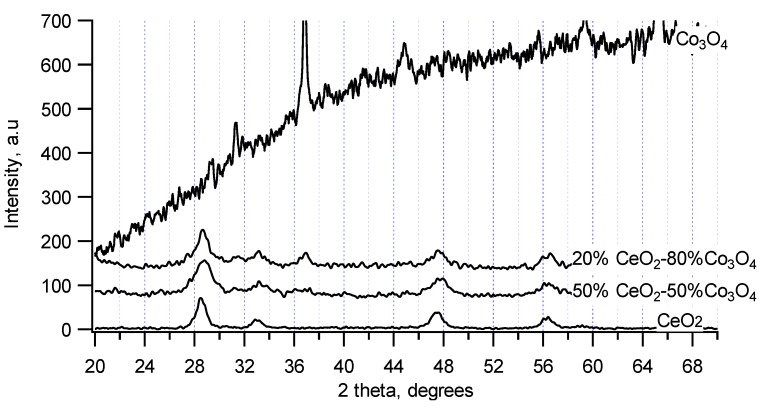
X-ray patterns of CeO_2_-Co_3_O_4_ chemical mixtures.

Chemical mixtures of CeO_2_ and Co_3_O_4_ were also studied since the results in Section 3.2 shows that Co_3_O_4_ exhibits high propylene conversion at low temperature although it did not exhibit high CO_2_ selectivity at high temperatures. Meanwhile, CeO_2_ exhibits high CO_2_ selectivity at high temperatures although its propylene conversion is not as high as that of Co_3_O_4_ at low temperatures. Therefore, when CeO_2_ and Co_3_O_4_ are mixed together, the obtained catalysts may exhibit high conversion of propylene at low temperatures and high CO_2_ selectivity at high temperatures. XRD patterns of some CeO_2_–Co_3_O_4_ chemical mixtures (20% and 50% mol of CeO_2_) in the comparison with XRD patterns of pure CeO_2_ and Co_3_O_4_ are presented in Figure 5. Pure Co_3_O_4_ synthesized at 550 °C exhibited a strong amorphous nature with a high and rough baseline but the strongest XRD reflections of a cubic Co_3_O_4_ structure (a, b, c parameters of 8.1 nm) were still detected at 2θ = 31°, 37°, 45°, 59° and 65°. Meanwhile, XRD patterns of CeO_2_–Co_3_O_4_ chemical mixtures (even up to 80% Co_3_O_4_) show the presence of mainly CeO_2_-like structure. However, there are shifts of ceria reflections to higher 2θ values and rougher baselines than that of pure CeO_2_. Thus, like the chemical mixtures of CeO_2_ and ZrO_2_, there may be also a formation of a solid solution in CeO_2_–Co_3_O_4_ chemical mixtures with the replacement of Co (atomic radius is 125 pm) for Ce (atomic radius is 181.8 pm) in the structure of CeO_2_. Because the content of Co_3_O_4_ was higher, Co_3_O_4_ phase may still be existed but as small or amorphous particles surround the solid solution of CeO_2_–Co_3_O_4_, leading to the rougher baselines than that of pure CeO_2_. The CeO_2_–Co_3_O_4_ chemical mixtures possess surface areas around 45 m^2^/g, which are higher than those of pure CeO_2_ (33 m^2^/g) and pure Co_3_O_4_ (11 m^2^/g). These may be reasons for the higher activity of the mixtures compared to pure components as described below. 

**Figure 6:**
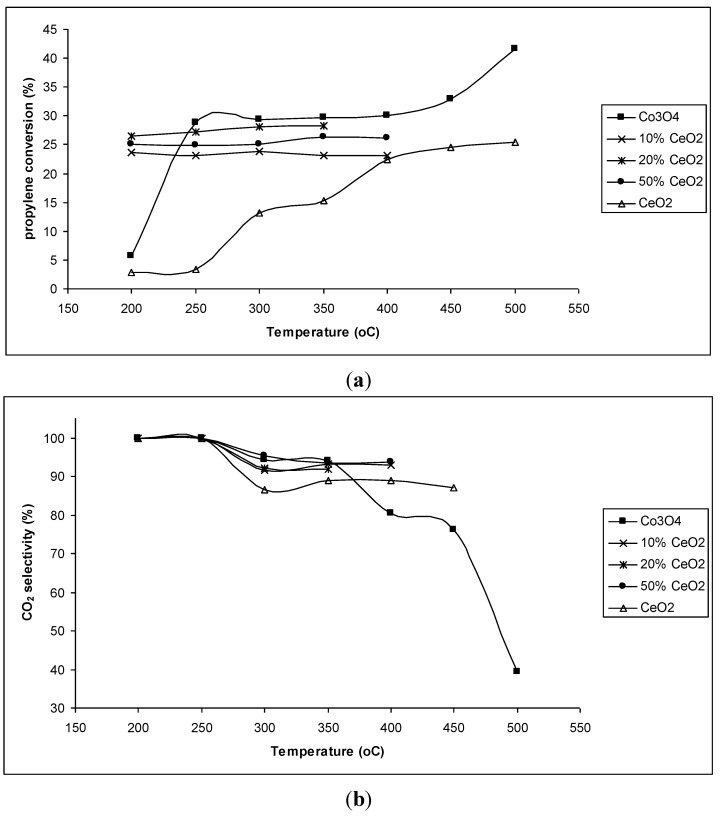
(**a**) Propylene conversion (%) and (**b**) CO_2_ selectivity (%) of CeO_2_–Co_3_O_4_ chemical mixtures at different reaction temperatures.

Propylene conversions and CO_2_ selectivity of CeO_2_–Co_3_O_4_ chemical mixtures are presented in Figure 6. Compared to the single oxides, CeO_2_–Co_3_O_4_ chemically mixed catalysts show high propylene conversions at lower temperature (200 °C). These chemical mixtures also possess as high CO_2_ selectivity as that of pure CeO_2_ at all reaction temperatures up to 400 °C. Under the oxygen sufficient condition (molar ratio of C_3_H_6_/O_2_ was 1/4), CeO_2_–Co_3_O_4_ chemical mixtures converted about 87% propylene since 250 °C with CO_2_ selectivity of about 98%. Under oxygen excess condition (molar ratio of C_3_H_6_/O_2_ was 1/5.5), CeO_2_–Co_3_O_4_ chemical mixtures converted 100% propylene since 250 °C with CO_2_ selectivity of 100%. Thus, the combination of Co_3_O_4_ with CeO_2_ improves propylene conversion and CO_2_ selectivity. Especially, this combination lowers the temperature of the maximum activity to 200 °C, which is very important for the treatment of hydrocarbon during the starting operation of the engines. However, the mechanical stability of Co_3_O_4_–CeO_2_ chemical mixtures is low. The materials were broken-up at temperatures higher than 400 °C. Therefore, Co_3_O_4_–CeO_2_ catalysts should be supported on high thermal resistant supports.

**Figure 7:**
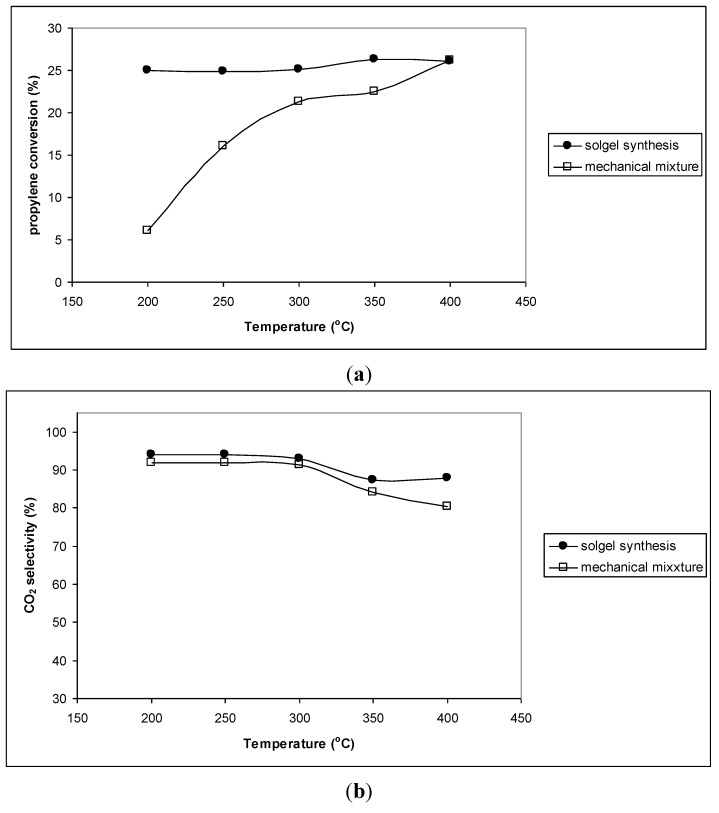
(**a**) Propylene conversion (%) and (**b**) CO_2_ selectivity (%) of the mixture containing 50 mol% CeO_2_ and 50 mol% Co_3_O_4_ synthesized by mechanical mixing and sol-gel method at different reaction temperatures.

The reason for the enhancement in catalytic activity of CeO_2_–Co_3_O_4_ chemical mixture samples may be the formation of the solid solution in the chemical mixtures of CeO_2_–Co_3_O_4_ resulted by the replacement of Co atoms for Ce atoms as seen from XRD patterns (Figure 5). To prove this assumption, the CeO_2_–Co_3_O_4_ mechanical mixture containing 50 mol% CeO_2_ was also tested for the reaction (Figure 7). It can be observed that both propylene conversion and CO_2_ selectivity of the mechanical sample are lower than that of the sol-gel prepared sample, where the formation of a solid solution was detected. Especially, like pure CeO_2_ and Co_3_O_4_, the mechanical mixture exhibited very low propylene conversion at low temperature (200 °C) while much higher propylene conversion had already been obtained on the chemical mixture at this temperature.

TPR–H_2_ data of pure CeO_2_, Co_3_O_4_ and the chemical mixture of 20% CeO_2_–80% Co_3_O_4_ in Table 3 helped to explain the activity of the mixtures and the pure oxides. TPR–H_2_ shows that Co_3_O_4_ exhibited an excellent mobility of oxygen as its consumed H_2_ quantity was highest amongst the investigated catalysts. Co_3_O_4_ was also reduced at lower temperatures than CeO_2_, which explains for the fact that Co_3_O_4_ exhibited good activity at lower temperature than CeO_2_. The chemical mixture of 20% CeO_2_–80% Co_3_O_4_ did not possess a larger quantity of mobile oxygen than pure Co_3_O_4_ but was reduced at lower temperature (since 279 °C); therefore, the chemical mixture of 20% CeO_2_–80% Co_3_O_4_ was able to convert propylene at lower temperature than Co_3_O_4_. For the chemical mixture of 20% CeO_2_–80% Co_3_O_4_, a solid solution was also formed as seen from XRD pattern of the sample, but the amount of consumed H_2_ of the mixture was not higher than that of pure Co_3_O_4_. Therefore, this mixture did not result in higher conversion of propylene than those of pure oxides; the advantage of this catalyst was combining well activity of both CeO_2_ and Co_3_O_4_ resulting in a catalyst with good activity at a wider temperature range. To explain for the fact that although the chemical mixture of 20% CeO_2_–80% Co_3_O_4_ possesses lower quantity of mobile oxygen than pure Co_3_O_4_ but there was no decrease in catalytic activity, a careful look on both TPR-H_2_ results and catalytic activities of good chemical mixtures of CeO_2_ with ZrO_2_ and Co_3_O_4_ also means something. It was seen that the chemical mixtures of CeO_2_ with ZrO_2_ (Ce_0.8_Zr_0.2_O_2_, Ce_0.9_Zr_0.1_O_2_) also exhibited good activity but the amount of consumed H_2_ was only about 30 mL/g while that of CeO_2_–Co_3_O_4_ chemical mixture was higher than 100 mL/g. Thus, it may be assumed that to ensure good oxidation of propylene, amount of mobile oxygen may be a certain value. If a catalyst possesses the amount of mobile oxygen higher than that necessary value, the activity may not increase significantly any more.

In order to explore the influences of co-existing gases and oxygen concentrations on catalytic performances, potential chemical mixtures of CeO_2_ with ZrO_2_ and Co_3_O_4_ (Ce_0.8_Zr_0.2_O_2_ and 20% CeO_2_ + 80% Co_3_O_4_) were studied in more details (Table 4). It is clear from Table 4 that these mixtures exhibited good activity for the oxidation of propylene not only under oxygen deficient condition but also under oxygen sufficient condition. Especially, under oxygen excess condition (condition 3 and 4), the chemical mixture of 20% CeO_2_ and 80% Co_3_O_4_ was able to convert 100% propylene and CO since 200 °C. A presence of 2% H_2_O did not influence significantly on the activity of this catalyst except that the minimum active temperature increases from 200 to 250 °C. The influence of CO and H_2_O was also not significant for the catalyst Ce_0.8_Zr_0.2_O_2_, proving that Ce_0.8_Zr_0.2_O_2_ and 20% CeO_2_–80% Co_3_O_4_ catalysts are stable catalyst for the oxidation of propylene under different reaction conditions. Compare to 20%CeO_2_-80% Co_3_O_4_ catalyst, Ce_0.2_Zr_0.2_O_2_ catalyst exhibited less activity under oxygen sufficient and excess conditions as conversion of propylene was less than and the temperature of the maximum activity was higher than those of 20% CeO_2_–80% Co_3_O_4_ catalyst. Catalytic activities of these catalysts are comparable to those of noble catalysts under the same deficient and sufficient conditions (at the same air to fuel ratios of 10 and 14) [27]. The catalyst mixtures of CeO_2_ and Co_3_O_4_ in this work even exhibited an advantage of having maximum activity at lower reaction temperature. The catalysts were stable during different catalytic cycles; the conversion and selectivity were almost unchanged during at least three catalytic cycles.

**Table 4:** The influences of co-existing gases (CO, H_2_O) and oxygen concentrations on catalytic activities (propylene conversion, %) of some potential chemical mixtures of CeO_2_ catalysts.

Temp. (°C)	Ce_0.8_Zr_0.2_O_2_				20% CeO_2_–80% Co_3_O_4_			
	1	2	3	4	1	2	3	4
200	3.52	1.47	-	3.02	26.58	7.2	100	1.4
250	4.43	-	-	4.05	27.16	86.87	100	99.80
300	12.39	4.12	-	6.02	28.03	87.27	100	100
350	28.79	11.27	6.99	15.91	28.28	87.83	100	100
400	30.07	25.97	19.69	51.74	-	86.95	100	100
450	30.18	59.52	49.91	81.27	-	86.73	100	100
500	30.80	85.42	90.67	89.08	-	87.08	100	100

1. Gas composition: 2.5% C_3_H_6_, 2.5% O_2_, N_2_ balance; 2. Gas composition: 0.9% C_3_H_6_, 4.1% O_2_, N_2_ balance; 3. Gas composition: 0.9% C_3_H_6_, 0.3% CO, 5% O_2_, N_2_ balance; 4. Gas composition: 0.9% C_3_H_6_, 0.3% CO, 2% H_2_O, 5% O_2_, N_2_ balance.

Mixtures of CeO_2_ and MnO_2_ were also expected to exhibit good activities since MnO_2_ was one of the most active catalysts as investigated in Section 3.2. However, this catalyst system requires a lot of detailed investigations in order to explain thoroughly their properties. Therefore, theses mixtures will be studied in a separate paper. 

## 4. Conclusions

The results of this work show that MnO_2_, CeO_2_ and Co_3_O_4_ are the most promising catalysts to convert propylene under oxygen deficient conditions. CeO_2_ not only shows a high propylene conversion but also a high CO_2_ selectivity at high temperatures. Therefore, CeO_2_ should be chosen as one component of the mixed catalysts for the treatment of propylene.

The catalytic properties of CeO_2_ could be improved by the combination with other components such as ZrO_2_ and Co_3_O_4_. Ce_0.9_Zr_0.1_O_2_ and Ce_0.8_Zr_0.2_O_2_ solid solutions exhibit the highest propylene conversions and CO_2_ selectivities. The chemical mixture of CeO_2_ and Co_3_O_4_ not only exhibits the highest propylene conversion and CO_2_ selectivity but also converts the largest amount of propylene at much lower temperatures compared to other catalysts. These catalysts also exhibited good activities when tested under oxygen sufficient and excess conditions and with the presence of co-existing gases (CO, H_2_O).
